# *Aeromonas salmonicida* binds α2-6 linked sialic acid, which is absent among the glycosphingolipid repertoires from skin, gill, stomach, pyloric caecum, and intestine

**DOI:** 10.1080/21505594.2022.2132056

**Published:** 2022-10-07

**Authors:** John Benktander, Henrik Sundh, Sinan Sharba, Susann Teneberg, Sara K. Lindén

**Affiliations:** a Department of Medical Biochemistry and Cell Biology, Institute of Biomedicine, Sahlgrenska Academy, University of Gothenburg, Gothenburg, Sweden; b Department of Biological and Environmental Sciences, University of Gothenburg, Gothenburg, Sweden

**Keywords:** Glycosphingolipid, Atlantic salmon, host–pathogen interaction, Aeromonas salmonicida, mucin, glycan, bacteria, epithelial surface, mucosa

## Abstract

Carbohydrates can both protect against infection and act as targets promoting infection. Mucins are major components of the slimy mucus layer covering the fish epithelia. Mucins can act as decoys for intimate pathogen interaction with the host afforded by binding to glycosphingolipids in the host cell membrane. We isolated and characterized glycosphingolipids from Atlantic salmon skin, gill, stomach, pyloric caeca, and intestine. We characterized the glycosphingolipids using liquid chromatography – mass spectrometry and tandem mass spectrometry and the glycan repertoire was compared with the glycan repertoire of mucins from the same epithelia. We also investigated *Aeromonas salmonicida* binding using chromatogram and microtiter well based binding assays. We identified 29 glycosphingolipids. All detected acid glycans were of the ganglio-series (unless shorter) and showed a high degree of polysialylation. The non-acid glycans were mostly composed of the neolacto, globo, and ganglio core structures. The glycosphingolipid repertoire differed between epithelia and the proportion of the terminal moieties of the glycosphingolipids did not reflect the terminal moieties on the mucins from the same epithelia. *A. salmonicida* did not bind the Atlantic salmon glycosphingolipids. Instead, we identified that *A. salmonicida* binding to sialic acid occurred to α2–6 Neu5Ac but not to α2–3 Neu5Ac. α2–6 Neu5Ac was present on mucins whereas mainly α2–3 Neu5Ac was found on the glycosphingolipids, explaining the difference in *A. salmonicida* binding ability between these host glycoconjugates. *A. salmonicida´s* ability to bind to Atlantic salmon mucins, but not the glycosphingolipids, is likely part of the host defence against this pathogen.

## Introduction

Natural fish stocks are overexploited and therefore aquaculture is an expanding food sector. Disease outbreaks in aquaculture facilities cause ethical and economical problems for the industry. In some countries, large amounts antimicrobials are used, leading to accumulation of antibiotic residues in the aquatic environment and favouring growth of resistant microorganisms [1]. The Atlantic salmon is one of the commercially most important fish species in aquaculture. Knowledge on the molecular basis for host protection versus infection mechanisms can contribute to the development of alternate methods for disease prevention in aquaculture.

Carbohydrates play an important role in the innate immunity of multicellular organisms. They can both protect against infection and act as targets promoting infection. Mucins, a major type of secreted mucosal proteins, are covered by glycans, and can act as decoys to prevent attachment to the epithelial surfaces by pathogens [2,3]. Mucins are major components of the continuously secreted mucus layer covering the fish epithelia and thus comprise the first contact point between pathogens and their hosts [4]. Glycosphingolipids (GSL), another type of glycoconjugate comprised of a lipid ceramide part and a carbohydrate part, are embedded in the outer layer of the cell membrane. GSL are involved in many functions, including cell signalling and the immune response [5] but can also be used as adhesion targets for pathogens, allowing for intimate interaction with epithelial cells to cause infection [6,7]. The downstream outcome resulting from the pathogen binding to mucins is most likely more beneficial to the host than if the pathogen binds to GSL.

Atlantic salmon mucin glycosylation has been relatively well studied, both with regard to differences in populations in different geographical regions and with regard to effects of stress and infection [8–10]. Atlantic salmon skin mucin *O*-glycans are relatively short with an average of 2–3 saccharide units and a maximum of eight saccharide units, while most gastrointestinal mucins have three to five saccharide units, but can be composed of up to 12 saccharides [10,11]. Furthermore, Atlantic salmon intestinal mucin glycans only carry the sialic acid *N*-Acetylneuraminic acid (Neu5Ac) whereas skin mucin *O*-glycans contain three different types of sialic acids: *N*-Glycolylneuraminic acid (Neu5Gc) and deaminoneuraminic acid (Kdn) [10,11]. Gill mucin *O*-glycans are of similar size as intestinal glycans, but have a high degree of fucosylation in the form of blood group like structures [8].

In contrast, GSL from Atlantic salmon have not been previously described and not much is known about fish GSL. However, a comprehensive study on zebrafish demonstrated structures with terminal Fucα1-3GalNAc and many gangliosides [12]. A study on rainbow trout liver has detected GSL with four neutral structures; glucosylceramide, galactosylceramide, globoside, and lactosylceramide. This study also demonstrated five acid GSLs: sulfatide, and the gangliosides GM2, GM3, GD1a, and 9-*O*-Acetyl GD3 [13].

*A. salmonicida* is a bacterium that causes morbidity and mortality in aquaculture. Sialic acids on Atlantic salmon mucins are a critical part of the epitope that *A. salmonicida* binds to [14]. *A. salmonicida* binding is affected by Ca^+2^ levels and fluid velocity [15,16]. Furthermore, *A. salmonicida* growth can be enhanced by *N*-acetylhexosamines, although capping of *N*-acetylhexosamine containing glycans by sialic acids can limit this effect [15]. It is currently not known if *A. salmonicida* interact with GSL.

The ability of mucins to act as decoys for the more intimate interaction with the host afforded by binding to GSL for a human pathogen, and that *A. salmonicida* can bind to Atlantic salmon mucins [2,3,6,7,14], lead us to hypothesize that the terminal epitopes on GSL are similar to the terminal epitopes on mucins from the same epithelia and that *A. salmonicida* therefore can bind to GSL too.

The aims of this study were to characterize the GSL repertoire of Atlantic salmon skin, gill, stomach, pyloric caeca, and intestine, to compare the glycans identified with glycans previously demonstrated to be present on mucins [10,11], and to investigate *A. salmonicida* binding to the GSLs.

## Methods

### Atlantic salmon rearing and sample collection

Atlantic salmon (parents: wild Göta Älvslax) were raised in freshwater (Långhults Lax AB, Långhult, Sweden). The fish were fed Biomar Inicio (2 mm; Biomar) daily in excess, including the day of sampling. Fourteen Atlantic salmon parr, approximately 20 g were sedated with MS-222 (20 mg/mL) and tissues harvested: skin from the body (i.e. not the head) was pulled off, while the gills, oesophagus and stomach, pyloric caeca, proximal and distal intestine were cut out (the gastrointestinal content was gently scraped out). The tissues were immediately placed in tubes on dry ice and kept at −80°C until use. The Ethical Committee for Animal Experiments in Gothenburg, Sweden, approved the use of the fish (#177/2013).

### GSL extraction and isolation

The method was performed as previously described [17]. Pooled tissue samples (See Table 1) stored at -80°C were lyophilized. From the dried samples the non-polar compounds were extracted by running 24 h in a Soxlet apparatus with a chloroform: methanol solution (2:1 by volume), then another 24 h in chloroform: methanol solution (1:9 by volume). The solutions were dried and pooled. By adding 10 ml/g of 0.2 M KOH in methanol, for a mild alkaline methanolysis the ester linkages of phosphoilipids were hydrolysed. Note that eventual ester linkage modifications and other non-alkaline stable modifications on the GSLs may be lost in this step. The solutions were dialysed against running tap water for at least 3 days, after adding chloroform and water to form a two-phase system in molecular membrane tubing (MWCO:12–14 kD, Spectra/Por). The dialysed solutions were dried and then subjected to a silicic acid column to separate the fatty acids and other non-polar compounds from the GSLs. Next, a column with DEAE (Diethylaminoethyl) ion-exchange resin was used to separate the GSL with acid moieties from the neutral. The acidic fractions were eluted with 5% LiCl in methanol, dialysed for at least three days and dried again. The neutral fractions were acetylated with a 1:1:1 (by volume) mixture of chloroform: pyridine: acetic acid anhydride. After addition of methanol the fractions were dried. The acetylated GSLs were then separated from the sphingomyeline based on polarity by using a silicic acid column. Following that, the acetylated glycosphingolipid fractions were deacetylated by adding 0.2 M KOH in methanol and samples dialysed in water. The dried deacetylated GSL fractions were chromatographed on DEAE ion-exchange columns separating any remaining acid GSLs from the neutral fractions. The acid GSL fractions separated with LiCl were pooled with the acid fraction from first DEAE column after dialysis. A final silicic acid column was run on the neutral fractions eluted from the DEAE column to further separate away remaining non-polar compounds.

**Table 1.** Yield of GSL isolated from 14 pooled Atlantic salmon (20 g each).

Species	Tissue	Dry weight (g)	Neutral GSLs (mg)	Acid GSLs (mg)	Neutral GSLs (mg)/tissue (g)	Acid GSLs (mg)/tissue (g)
Atlantic salmon	Skin	6.15	16.7	27.3	2.7	4.4
Atlantic salmon	Gills	1.53	5.2	12.3	3.4	8.0
Atlantic salmon	Esophagus+Stomach	1.49	13.3	21.9	8.9	14.7
Atlantic salmon	Pyloric caeca	3.13	6.9	8.9	2.2	2.8
Atlantic salmon	Intestine	2.26	16.2	62.3	7.2	27.6

### Liquid chromatography – mass spectrometry (LC – MS)

Preparation of the neutral glycosphingolipids for LC-MS was done as previously described [18]. *Rhodococcus* spp. endoglycoceramidase II (Takara Bio Europe, Gennevilliers, France) was utilized to hydrolyse neutral GSLs. Fifty μg of the GSLs were diluted in 100 μl 0.05 M sodium acetate buffer, pH 5.0, with 120 μg sodium cholate. After being mixed thoroughly, endoglycoceramidase II (1 mU) was added and samples put in 37°C. After 48 h, chloroform/methanol/water (final proportions by volume: 8:4:3) was added. The cleaved off oligosaccharide-containing upper phase was desalted with Sep-Pak QMA cartridges (Waters, Milford, MA), dried under flowing N_2_ and diluted in 50 µl water before LC-MS. The oligosaccharides released from the GSLs were eluted from a 10 cm × 250 μm column containing 5 μm porous graphitized carbon (PGC) particles (Thermo Scientific, Waltham, MA, USA), using a 0–40% gradient of acetonitrile in 10 mM ammonium bicarbonate at a flow rate of 10 μL/min over 40 min. The acidic GSLs were run in native state. The GSLs were dissolved in methanol:acetonitrile 75:25 (by volume) and separated on a 200 × 0.150 mm polyamine II (5 μM particles) column (YMC Europe GmbH, Dinslaken, Germany). The GSLs were eluted using a gradient of 0–50% water with 10 mM ammonium bicarbonate with a flow rate of 3 μL/min over 40 min.

Both the GSL derived neutral oligosaccharides and the native acidic glycans were analysed in negative-ion mode using an LTQ mass spectrometer (Thermo Scientific) as previously described [11], with the following conditions: electrospray voltage: 3.5 kV; capillary voltage: −33.0 V; capillary temperature: 300°C; sheath gas: compressed air. For the LC – MS; full scans were performed in the mass range *m*/*z* 380–2000. MS/MS; minimal signal 300 counts; normalized collisional energy 35%; activation time 30 ms; isolation width *m*/*z* 2.0. The Xcalibur software (version 2.0.7, Thermo Scientific) was used for data analysis, glycans were manually annotated from their MS/MS spectra and, when possible, validated by the Unicarb-DB [19] database.

### Immunofluorescence

Deparaffinized and rehydrated tissue sections from Atlantic salmon skin, pyloric ceaca, and gills were heated (99°C) for 10 min in 0.01 M citric acid buffer, pH 6. Sections were immersed in 5% foetal bovine serum (FBS) diluted in phosphate-buffered saline (PBS) for 1 h and then incubated with an anti-sulfatide antibody (MAB1326, monoclonal mouse anti-O4, R&D systems) diluted (1:100) in 5% FBS diluted in PBS at 4°C overnight. The following day, slides were rinsed in PBS containing Tween20 (0.05%) and incubated with Alexa Flour 488-conjugated goat anti-mouse IgG (ab150113, Abcam) diluted (1:1500) in 5% FBS diluted in PBS at room temperature (RT) for 1 h. Subsequently, slides were rinsed in PBS and incubated with CellMask (C10046, Thermo Fisher Scientific) diluted (1:14000) in PBS at RT for 30 min to outline the tissue. Finally, sections were rinsed in PBS and mounted with DAPI with prolong Gold anti-fade (P36935, Thermo Fisher Scientific).

### Bacteria culture conditions

*A. salmonicida* subspecies *salmonicida* strain VI-88/09/03175 (culture collection, Central Veterinary Laboratory, Oslo, Norway) were brought up from −80°C and cultured at 16 °C on tryptic soy agar plates for 48 h.

### Chromatogram binding assay

Chromatogram binding assays were performed as previously described [20]. 35S-methionine (50 μCi, PerkinElmer; NEG77207MC) in 0.5 ml PBS, pH 7.3, were added to the *A. salmonicida* culture plates to radiolabel the bacteria. After incubation for 16–24 h, the bacteria were harvested, centrifuged three times and diluted to 1 × 10^8^ CFU/ml in PBS containing 2% bovine serum albumin (w/v), 0.1% NaN_3_ (w/v), and 0.1% Tween20 (by volume). The suspensions had approximately 1 cpm per 100 bacteria.

Thin-layer chromatograms were made by running the GSLs in a mixture of chloroform:methanol:water (by volume: 60:35:8). Chromatograms were dried and subjected to diethylether/*n*-hexane (by volume: 1:5) containing 0.5% (w/v) polyisobutylmethacrylate (Sigma-Aldrich; 181544). The chromatograms were soaked in PBS containing 2% bovine serum albumin (w/v), 0.1% NaN_3_ (w/v), and 0.1% Tween20 (by volume) for 2 h at RT after drying. The chromatograms were then covered with radiolabeled *A salmonicida* (2–5 × 10^6^ cpm/ml) diluted in the same solution, incubated for 2 h at RT and repeatedly washed with PBS. XAR-5 X-ray films (Carestream; 8941114) were exposed to the chromatograms for 12–48 h for detection of radiolabeled bacteria.

*A. salmonicida binding to sialic acids on human serum albumin (HSA) conjugates*

*A. salmonicida* binding to 3´-Sialolactose-acetyl-phenylenediamine (ADP) HSA conjugate and 6´-Sialolactose-ADP HSA conjugate (IsoSep, Tullinge, Sweden) coated at 8 µg/ml onto Nunc 96-well polysorp plates (ThermoFisher Scientific, Roskilde, Denmark) was analysed as previously described for other glycoconjugates [21].

### Statistics

Data was plotted using Graphpad Prism and significance was calculated using Student’s-t test. A *p*-value above 0.05 was considered statistically significant.

## Results

GSL isolation

Tissues from 14 fish with an average size of 20 g were pooled and neutral and acid GSLs were isolated (See Table 1). The respective weights of the isolated fractions revealed that the proportion of acid GSLs was higher than neutral GSLs in skin, gill, stomach, pyloric caeca and intestine, with the intestine having the highest proportion of acid GSLs (Table 1).

LC-MS of neutral GSLs

**Figure 1.**
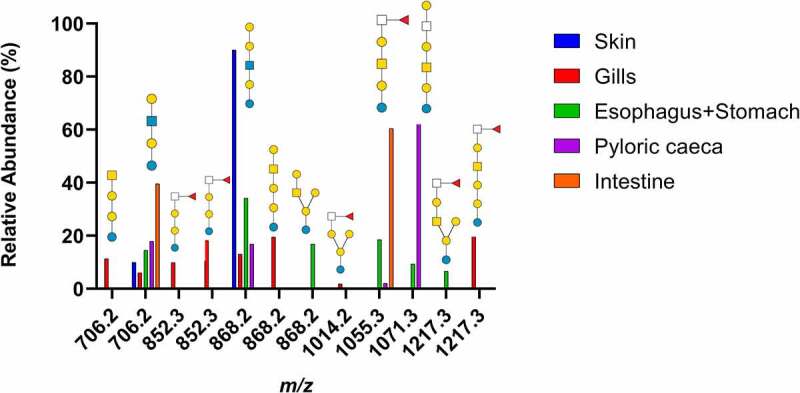
Summary of neutral GSL found in Atlantic salmon epithelia. Symbol explanation: yellow square=*N*-acetylgalactosamine (GalNac), yellow circle=galactose (Gal), blue square=*N*-acetylglucosamine (GlcNac), blue circle=glucose (Glc), white square=*N*-acetylhexosamine (HexNac) and red triangle=fucose (Fuc).

After using recombinant endoglycoceramidase II enzyme to cleave the ceramide from the glycan part of the purified neutral GSLs, the glycans were examined by LC-MS using a porous graphitized carbon column. MS/MS was used to identify the glycan structures. The results are summarized in Figure 1 and Supplementary Table 1.

**Figure 2.**
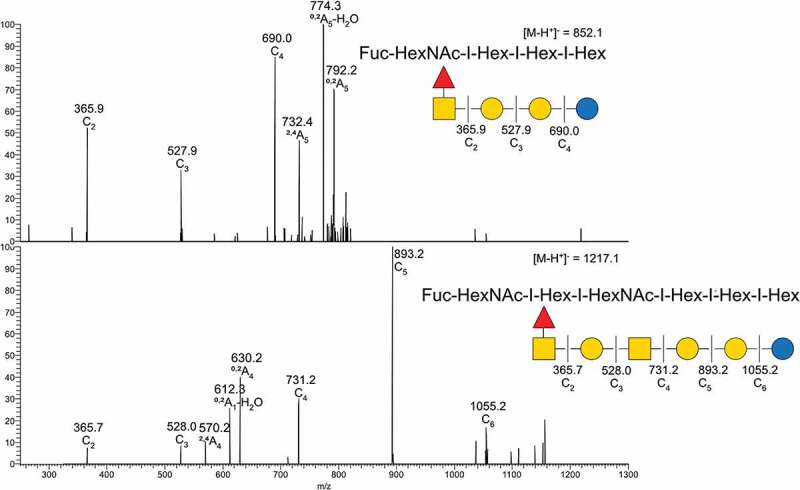
MS/MS interpretation of two fucosylated structures from Atlantic salmon gills. **A**) *m/z* 852 suggest a fucosylated globo structure. **B)** *m/z* 1217 suggest an elongated fucosylated globo structure with a 1–4 linkage between the terminal end hexose and middle HexNac giving the ^0,2^A_4_ cross ring fragment. Symbol explanation: yellow square=*N*-acetylgalactosamine (GalNac), yellow circle=galactose (Gal), blue circle=glucose (Glc), blue square=*N*-acetylglucosamine (GlcNac), white square=*N*-acetylhexosamine (HexNac) and red triangle=fucose (Fuc).

From the base-peak chromatogram from ESI-MS, iso-globo type structures (Galα1–3 Galα1-4Glc) but also ganglio (Galß1-3GalNAcß1–4 Galα1-4Glc) and branched hybrids of them (Galß1-3GalNAcß1-4[Galα1-3]Galα1-4Glc) were found. In addition, the presence of neo-lacto type structures (Galß1-4GlcNAcß1–3 Galα1-4Glc) were identified (Figure 1). Fucosylated structures linked to terminal HexNAcs were found (Figure 2), with the highest relative abundance of fucosylated structures found in the gills and intestine (Figure 1).

### LC-MS of acid GSLs

**Figure 3.**
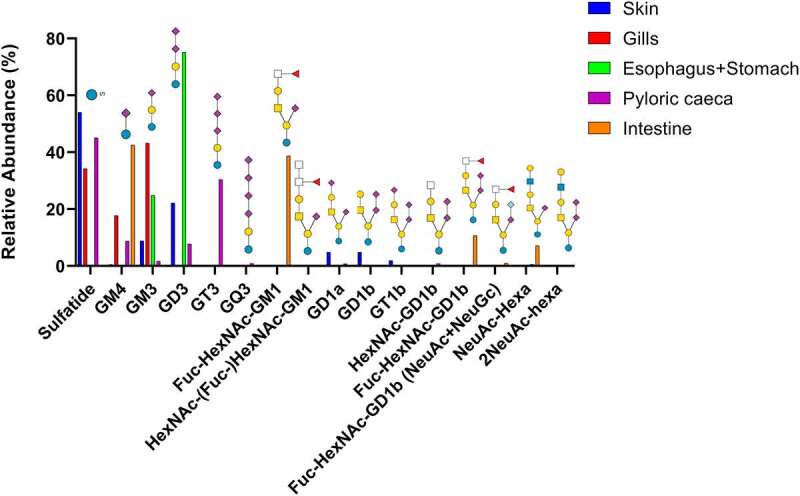
Summary of acid GSL found in Atlantic salmon epithelia. Symbol explanations: yellow square=*N*-acetylgalactosamine (GalNac), yellow circle=galactose (Gal), blue square=n-acetylglucosamine (GlcNac), blue circle=glucose (Glc), white square=*N*-acetylhexosamine (HexNac), red triangle=fucose (Fuc), purple diamond= *N*-acetylneuraminic acid (Neu5ac) and S=sulphate group.

Native acid GSLs were run on LC-MS using a polyamine II (YMC, Kyoto, Japan) column packed in-house, with MS/MS used for glycan structure identification. Results are summarized in Supplementary Table 1 and Figure 3.

**Figure 4.**
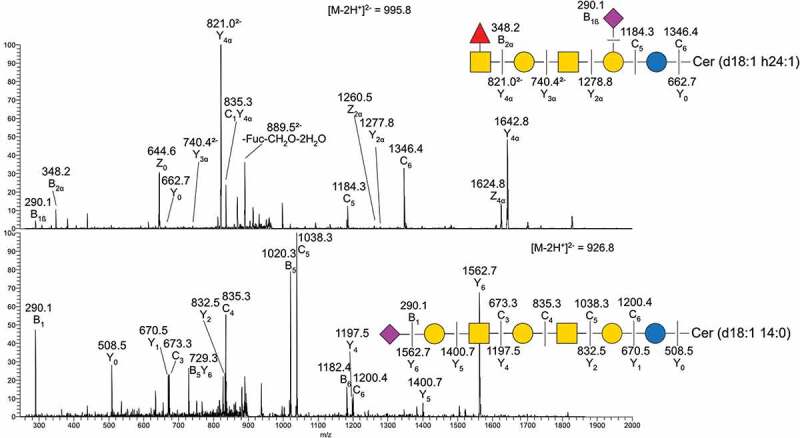
MS/MS of two native acid GSL from Atlantic salmon intestine. **A)** MS/MS of *m/z* 995.8 **B)** MS/MS of *m/z* 926.8. Symbol explanations: yellow square=*N*-acetylgalactosamine (GalNac), yellow circle=galactose (Gal), blue circle=glucose (Glc), red triangle=fucose (Fuc) and purple diamond= *N*-acetylneuraminic acid (Neu5ac).

The identified GSLs were of the ganglio-series with the vast majority having Neu5Ac, and a small amount of Neu5Gc. Sulfatides were also found in skin, gills, and pyloric caeca, but not in the esophagus/stomach or the intestine. Compounds with one or more Neu5Acα2-8Neu5Ac-X were common, with the highest relative abundance in stomach (47%) and the lowest relative abundance in intestine (15%, Figure 3). Sialylation occurred both at the inner lactose unit and the terminal galactose with α2–3 linkages, demonstrated by a lack of ^0,2^X-fragments (Figure 4).

#### Salmonicida binds to α2–6 linked Neu5Ac

**Figure 5.**
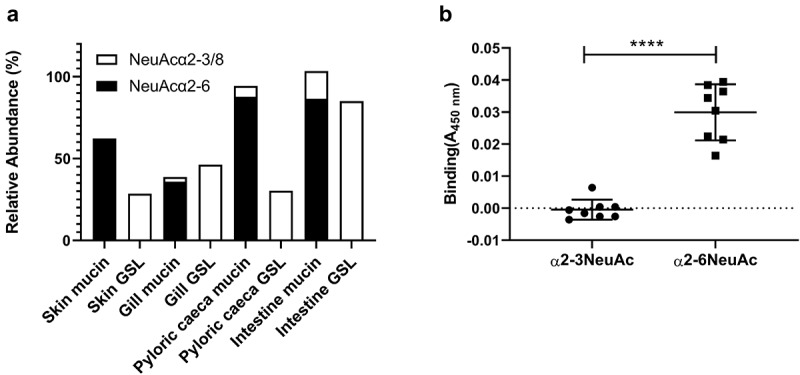
Distribution and *A. salmonicida* binding to α2–3 and α2–6 linked Neu5Ac. **A)** the relative abundance of α2–3 and α2–6 linked Neu5Ac on mucins and GSLs. Note that some glycan structures contain both α2–3 and α2–6 linked sialic acid. **B)** Binding of *A. salmonicida* to α2–3 and α2–6 sialyl-lacto HSA conjugates (8 µg/ml). No binding to α2–3 linked sialic acid was detected when compared to control (PBS), while the α2–6 linked sialic acids showed significant binding (n = 8). Data are shown after subtracting the average background control signal. Significance was calculated using Student’s-t test (****=p < 0.0001). Outliers in the background control (blank) were removed by ROUT (Q = 1%).

We examined *A. salmonicida* binding to both neutral and acid glycolipids isolated from all salmon epithelial sites using a chromatogram binding assay. However, in spite of performing the experiment three times we did not find any clear binding (data not shown). Since previous studies have demonstrated that sialic acid containing structures are important for *A. salmonicida* binding to mucins [14,15], we hypothesized that the difference in sialic acid linkages between the GSLs and mucin glycans may explain the results. The sialic acids on mucins were mainly α2–6 linked, whereas α2–3 and the polysialic acid α2–8 linked sialic acids dominated in the GSLs [8,11] (Figure 5A). By investigating binding to reference Neu5Acα2-6-neolactotetra GSL, a weak band was observed on the film (data not shown). We therefore analysed *A. salmonicida* binding to α2–3 and α2–6 linked Neu5Ac-lactose HSA conjugates. In line with the binding to mucins and lack of binding to GSLs, the *A. salmonicida* did not bind to the α2–3 Neu5Ac-lactose HSA whereas it bound to α2–6 Neu5Ac-lactose HSA (Figure 5B).

### Comparison of mucin O-glycans with GSL glycans

**Figure 6.**
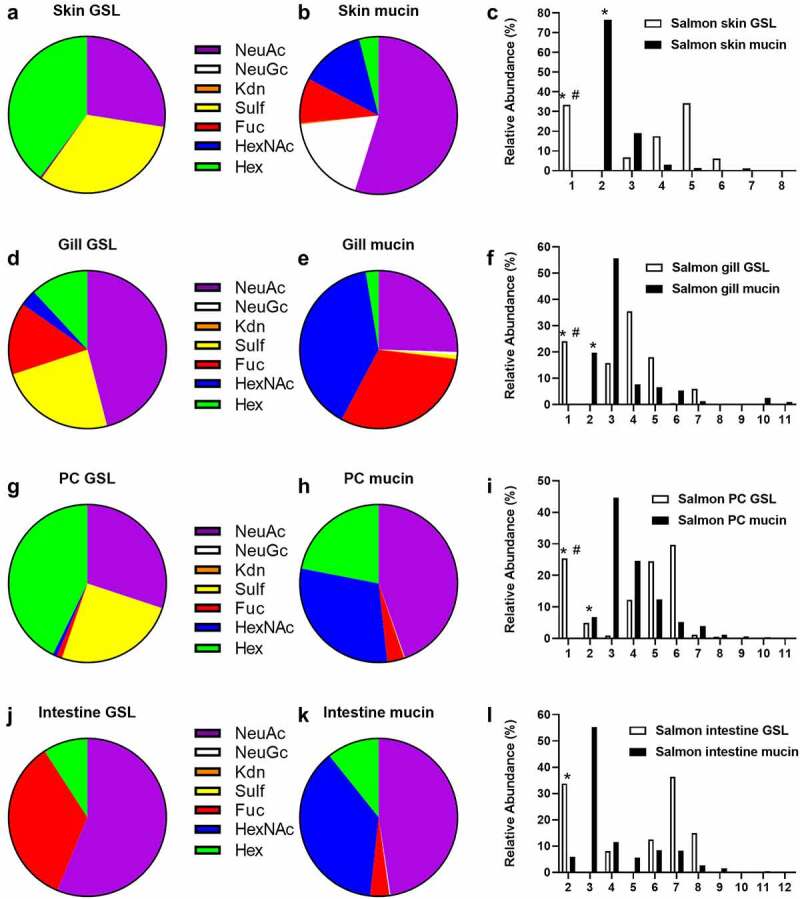
**Comparison of terminal epitopes and size between mucins and GSLs. A, B, D, E, G, H, J and K)** Comparison of terminal epitopes from GSL and mucin *O*-glycans. The acid and neutral GSL data were proportionally combined based on isolation data from table 1. The data was calculated based on the weight of the GSL (neutral/acid GSL) and number of terminal moieties. **C, F, I and L)** Comparison of number of monosaccharides in the GSL or *O*-glycans from the same organ tissue, based on previously published data [8–11]. *neutral GSL containing one or two monosaccharides could not be included due to having too small mass for MS detection. ^#^ Mucin glycans containing a single monosaccharide can not be detected either.

Since a likely function of the secreted mucins is to act as decoys for the more intimate interaction with the host afforded by binding to GSL, the GSL glycans were compared with the previously published [8–11] mucin *O*-glycan structures from the same epithelial site (excluding stomach, for which there is no mucin *O*-glycan data available). The number of observed glycans from the GSLs was lower than what was observed in mucins. The size of the majority of the detected glycans were larger in the GSLs than on mucins, however since the neutral GSLs only were detected when they have a larger than two saccharide unit length, the small size GSL are underrepresented in this study (Figure 6 C, F, I and L). No neutral GSL with one or two saccharide units were detected due to having less than 382 *m/z*, which was the minimum detection range of the mass spectrometer.

**Figure 7.**
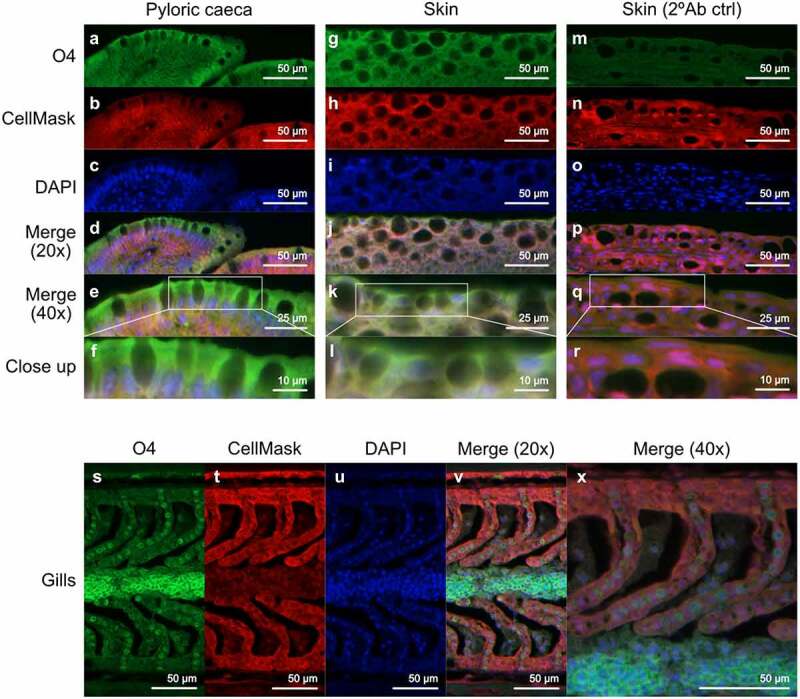
**Location of sulfatide in Atlantic salmon pyloric caeca, skin and gills**. (A, G and S) sulfatide tissue localization was visualized with the O4 antibody (green). (B, H, N and T) the tissue was outlined by CellMask (red). (C, I, O and U) DAPI (blue) stained the nuclei. (D, J, P and V) show the channels merged at 20x and (E, K, Q and X) at 40x. (F, L and R) Show close up images of the regions enclosed by white boxes in (E, K, Q and X). (*M*-R) show skin tissue without the O4 antibody, used as a background control.

Terminal HexNAc were rare on the GSLs, while quite prevalent in the mucins. Terminal hexose were prevalent on GSLs but present in lower amounts on mucins. Fucosylated glycans were prevalent in both GSLs and mucins, but the relative abundance of fucosylated structures differed between the epithelia. Overall, the terminal moieties on mucins and glycolipids did not match within the epithelia. For example, the gills was the site with the lowest mucin sialylation whereas it was the epithelial site with the second highest GSL sialylation, and the intestine was a site with low mucin fucosylation whereas it was the epithelial site with the second highest GSL fucosylation (Figure 6). Sulphation was prevalent (>25% of the structures) on GSLs in all epithelia except stomach and intestine, whereas sulphation was barely detected on the mucins. However, when staining tissue sections to determine which cells the sulfatides were present in, sulfatides were prevalent in epithelial cells from skin and pyloric caeca, but mainly present on red blood in the gills (Figure 7).

In the skin, a high level of sulphation was detected in the GSLs while none was found in the mucins. Instead, the skin mucins carried a high amount of terminal Neu5Gc, which resulted in that the proportion of glycans that were acidic was approximately 70% on both types of glycoconjugates.

The gills showed a high degree of fucosylation in both mucin and GSLs but the GSLs had more acid monosaccharides with sulphates and sialic acid whereas the mucin glycans were predominantly neutral.

For the pyloric caeca both sources show a low level of fucosylation and relatively high presence of terminal hexoses. Roughly half of the glycans were acid for both glycoconjugates, however approximately half of this was composed of sulfatides in the GSLs whereas it was all Neu5Ac on the mucins.

The intestinal GSLs carried a very high level of terminal fucosylation while fucosylation was modest in the mucins, which instead had terminal HexNAc. Roughly half of the glycans were sialylated in both mucin and GSLs with no sulphation detected.

## Discussion

Here, we characterized GSLs from Atlantic salmon skin, gill, stomach, pyloric caeca and intestine and found that the GSL repertoire differed between the epithelia. Furthermore, the terminal moieties of the GSLs did not closely reflect the terminal moieties on the mucins from the same epithelia, suggesting that pathogen interactions with these glycoconjugates differ. In contrast to the previously shown sialic acid dependent *A. salmonicida* binding to mucins, *A. salmonicida* did not bind to the Atlantic salmon GSLs in spite of them carrying sialic acid. Subsequently, we identified that *A. salmonicida* binding to sialic acid occurred to α2–6 Neu5Ac, but not to α2–3 Neu5Ac. α2–6 Neu5Ac was present on mucins, whereas mainly α2–3 Neu5Ac was found on GSLs, explaining the difference in binding ability between these glycoconjugates.

Since GSL isolation is a very time consuming process and require a large amount of starting material, less information is often available compared to other glycoconjugates. So far, only a couple of GSL studies on fish have been published, and none of these on Atlantic salmon epithelia [12,13]. We examined the glycosphingolipid repertoire in five different tissues: skin, gills, stomach, pyloric caeca and intestine. By LC-MS and tandem MS we identified 29 GSLs. All detected acid glycans were of the ganglio series (unless shorter) and showed a high degree of polysialylated glycans. The non-acid glycans were mostly composed of the neolacto, globo, and ganglio core structures. We compared the GSL glycans with glycans we previously identified on mucins from the same epithelia using the same methodology. The average length of the saccharide chain of the GSLs were longer than on mucins from the same tissue, but glycans with more than 8 saccharide units were not detected, compared to the mucins which has a low abundance of glycan chain comprised by up to 12 saccharide units among the *O*-glycans. In general, the differences between the mucin and GSLs terminal moieties were shown to be the higher degree of HexNAc terminating glycans in the mucin *O*-glycans, while GSLs more often had galactose terminating glycans. The sulphation was virtually absent from mucins, while present on GSLs from skin, gills, and pyloric caeca. However, this does not necessary mean that it is present on the cell surface. Barone *et al*. identified sulfatides from human embryonic stem cells, but they were not present on the cell surface, instead they were located in the mitochondria, ER, and Golgi [22]. In tissue sections, we identified that sulfatides were present in, and possibly on, epithelial cells in skin and pyloric caeca. In the gills, sulfatides were mainly present on the red blood cells, suggesting they are not available for interactions with microbes in the healthy gill.

The fucosylation of Atlantic salmon GSLs display similar terminals as mucins, with fucose linked to HexNAc or the elongated version HexNAc-(Fuc-)HexNAc-X. These type of structures have previously been shown to occur with a high degree of inter-individual variation in gill mucins [23]. Compared to mammals, the inter-individual diversity of Atlantic salmon mucin glycans is low [10,11,24,25]. These fucosylated structures could be a manner of diversifying the mucin glycan repertoire in the gill, in line with that the epithelial glycan diversity in humans is considered a population-based defence against pathogens [26]. Furthermore, these structures are similar to Lewis epitopes, both having fucosylated HexNAcs, which have important roles in infection and immunology in human epithelia [26,27]. The most prevalent GSL fucosylation was found in gills and intestine. In the mucins, the gills also showed a high degree of fucosylation while the intestinal mucins did not contain a high degree of fucosylation.

The results from the salmon GSLs show that the acid GSLs are the same types that are found in other mammals. This suggests that the ganglioside synthesis pathway present in salmon is similar to that of mammals [28]. As for the neutral salmon GSL, a similar case was seen there with the core structures also found in mammals [29–31]. The difference is that the commonly found blood group antigens such as the ABO and Lewis antigens are absent in the salmon, which instead has terminal fucosylated HexNAcs.

We found that *A. salmonicida* did not bind to α2–3 linked sialic acid but to α2–6 linked sialic acid (at least when the epitope is part of lactose HSA conjugates). This conclusion is further supported by absence of binding to the acid GSL fractions that contain no α2–6 linked sialic acids, while mucins from these epithelia, which carry α2–6 linked sialic acids, previously have been found to bind *A. salmonicida* [14,15]. These mucins display a large difference compared to GSLs with the mucins varying, depending on individual and tissue source, between 20% and 90% relative abundance of α2–6 linked sialic acid containing glycans. This may further suggest that the α2–6 linked sialic acids in the mucin *O*-glycans are used as decoys preventing adhesion to the membrane.

In conclusion, the terminal epitopes on the Atlantic salmon GSL glycans had both similarities and differences with the terminal glycan epitopes on mucins from the same epithelial site, and the differences were sufficiently pronounced to lead to differences in binding with the pathogen *A. salmonicida*. Weather the pathogen binds to GSL or mucins most likely have major effects on the downstream result of the host–pathogen interaction. The decoy function of mucins could be why a larger number of *O*-glycan structures are found on the mucins, compared to GSL. The lack of ability of *A. salmonicida* to bind to GSL, indicate that it is unlikely that *A. salmonicida* invades Atlantic salmon epithelial cells using GSL, however, other pathogens may use GSL for this purpose.

## Supplementary Material

Supplemental Material

## Data Availability

Raw mass spectrometry data files are available at http://doi.org/10.50821/GLYCOPOST-GPST000187.
