# Supplementary material for: Aeromonas salmonicida binds α2-6 linked sialic acid, which is absent among the glycosphingolipid repertoires from skin, gill, stomach, pyloric caecum, and intestine
Source: Virulence. 2022 Oct 7;13(1):1741–51. doi: 10.1080/21505594.2022.2132056 (PMC9553145; doi:10.1080/21505594.2022.2132056)
Supplement: Supplemental Material [file KVIR_A_2132056_SM1884.zip › supplementary/SI.pdf]

			Atlantic salmon Skin		Atlantic salmon Gills		Atlantic salmon Stomach+Esophagus		Atlantic salmon Pyloric caeca		Atlantic salmon Intestine	
[M-H+]-	m/z	Tentative structure	RT	Relative abundance (%)	RT	Relative abundance (%)	RT	Relative abundance (%)	RT	Relative abundance (%)	RT	Relative abundance (%)
706.2	706.2	GalNAc-Gal-Galα1-4Glc	nd	0.0	15.6/16.6	11.3	nd	0.0	nd	0.0	nd	0.0
706.2	706.2	Galß1-4GlcNAcß1-3Galα1-4Glc	19.8*	10.0	19.7	6.0	19.7*	14.5	19.7	17.9	19.8*	39.6
852.3	852.3	Fuc-HexNAc-Gal-Galα1-4Glc	nd	0.0	17.0/17.3	9.9	nd	0.0	nd	0.0	nd	0.0
852.3	852.3	Fuc-HexNAc-Gal-Galα1-4Glc	nd	0.0	18.7	18.2	nd	0.0	nd	0.0	nd	0.0
868.2	868.2	Gal-GalNAc-(Gal-)Galα1-4Glc	nd	0.0	nd	0.0	13.8/14.4*	16.9	nd	0.0	nd	0.0
868.2	868.2	Gal-GalNAc-Gal-Galα1-4Glc	nd	0.0	17.5/18.1	19.5	nd	0.0	nd	0.0	nd	0.0
868.2	868.2	Gal-Galß1-4GlcNAcß1-3Galα1-4Glc	22.8/23.3*	90.0	22.7/23.1	13.0	22.6/23.3	34.2	22.6/23.3	16.9	nd	0.0
1014.2	1014.2	Fuc-HexNAc-Gal-(Gal-)Gal-α1-4Glc	nd	0.0	21.3/21.7*	1.8	nd	0.0	nd	0.0	nd	0.0
1055.3	1055.3	Fuc-HexNAc1-3Galß1-3GalNAcß1-4Galα1-4Glc	nd	0.0	nd	0.0	18.2/19.0*	18.5	19.1*	2.1	19.0*	60.4
1071.3	1071.3	Gal-HexNAc-Galß1-4GalNAc-Gal-Galα1-4Glc	nd	0.0	nd	0.0	26.1/26.7*	9.3	26.1/26.7	63.1	nd	0.0
1217.3	1217.3	Fuc-HexNAc-Galß1-3GlcNAc-(Gal-)Galα1-4Glc	nd	0.0	nd	0.0	16.2/16.6*	6.6	nd	0.0	nd	0.0
1217.3	1217.3	Fuc-HexNAc-Galß1-4GalNAc-Gal-Galα1-4Glc	nd	0.0	22	20.2	nd	0.0	nd	0.0	nd	0.0

*Identified from MS1 and retention time
