# Supplementary material for: Aeromonas salmonicida binds α2-6 linked sialic acid, which is absent among the glycosphingolipid repertoires from skin, gill, stomach, pyloric caecum, and intestine
Source: Virulence. 2022 Oct 7;13(1):1741–51. doi: 10.1080/21505594.2022.2132056 (PMC9553145; doi:10.1080/21505594.2022.2132056)
Supplement: Supplemental Material [file KVIR_A_2132056_SM1884.zip › supplementary/SII.pdf]

	Ceramide			Atlantic salmon Skin		Atlantic salmon Gills		c salmon Stomach+Eso		antic salmon Pyloric ca		tlantic salmon Intestin	
m/z	Sphingosin	Fatty acid	Supposed structure	RT	Relative ab	RT	Relative ab	RT	Relative ab	RT	Relative ab	RT	Relative abundance (%)
750.8	d18:1	14:0	SO3-Galß1-Cer	3.0	3.4	nd	0.0	nd	0.0	1.8	33.3	nd	0.0
888.8	d18:1	24:1	SO3-Galß1-Cer	5.0	34.7	0.6	31.6	nd	0.0	4.4	11.8	nd	0.0
902.8	d18:1	25:1	SO3-Galß1-Cer	6.0	5.5	nd	0.0	nd	0.0	nd	0.0	nd	0.0
904.8	d18:1	h24:1	SO3-Galß1-Cer	6.0	10.5	2.4	2.6	nd	0.0	nd	0.0	nd	0.0
977.7	d18:1	h14:0	NeuAcα2-3Galß1-Cer	18.1	0.1	nd	0.0	nd	0.0	nd	0.0	13.6	22.9
1005.8	d18:1	h16:0	NeuAcα2-3Galß1-Cer	18.1	0.1	nd	0.0	nd	0.0	12.6	0.9	13.8	10.0
1115.8	d18:1	h24:1	NeuAcα2-3Galß1-Cer	17.9	0.3	nd	0.0	nd	0.0	12.6	7.2	13.1	7.6
1129.8	d18:1	h25:1	NeuAcα2-3Galß1-Cer	nd	0.0	nd	0.0	nd	0.0	12.6	0.3	13.1	2.0
1131.8	d18:1	h25:0	NeuAcα2-3Galß1-Cer	nd	0.0	nd	0.0	nd	0.0	12.5	0.3	nd	0.0
1123.7	d18:1	14:0	NeuAcα2-3Galß1-4Glcß1-Cer	20.2	0.8	10.3	3.3	10.2	4.4	13.1	1.0	nd	0.0
1151.7	d18:1	16:0	NeuAcα2-3Galß1-4Glcß1-Cer	20.2	0.9	10.4	3.6	nd	0.0	nd	0.0	nd	0.0
1261.8	d18:1	24:1	NeuAcα2-3Galß1-4Glcß1-Cer	20.2	4.4	10.4	5.9	11.4	13.8	13.2	0.4	nd	0.0
1277.8	d18:1	h24:1	NeuAcα2-3Galß1-4Glcß1-Cer	20.8	2.7	10.4	4.9	nd	0.0	13.3	0.3	nd	0.0
1279.8	d18:1	h24:0	NeuAcα2-3Galß1-4Glcß1-Cer	20.7	2.0	10.4	4.9	11.8	6.6	nd	0.0	nd	0.0
707.2/1415.7	d18:1	14:0	NeuAcα2-8NeuAcα2-3Galß1-4Glcß1-Cer	21.8	3.6	nd	0.0	nd	0.0	nd	0.0	nd	0.0
721.2/1442.7	d18:1	16:0	NeuAcα2-8NeuAcα2-3Galß1-4Glcß1-Cer	22.0	1.0	nd	0.0	11.7	15.4	nd	0.0	nd	0.0
729.1/1458.7	d18:1	h16:0	NeuAcα2-8NeuAcα2-3Galß1-4Glcß1-Cer	nd	0.0	nd	0.0	nd	0.0	13.7	2.1	nd	0.0
776.2/1552.8	d18:1	24:1	NeuAcα2-8NeuAcα2-3Galß1-4Glcß1-Cer	22.3	11.8	11.2	28.0	11.8	27.6	13.6	1.1	nd	0.0
784.2/1568.8	d18:1	h24:1	NeuAcα2-8NeuAcα2-3Galß1-4Glcß1-Cer	22.3	5.7	nd	0.0	11.7	15.8	13.7	3.2	nd	0.0
785.2/1570.8	d18:1	h24:0	NeuAcα2-8NeuAcα2-3Galß1-4Glcß1-Cer	nd	0.0	11.2	15.1	11.7	16.3	nd	0.0	nd	0.0
792.1/1584.7	d18:1	h25:0	NeuAcα2-8NeuAcα2-3Galß1-4Glcß1-Cer	nd	0.0	nd	0.0	nd	0.0	13.7	1.3	nd	0.0
874.5	d18:1	h16:0	NeuAcα2-8NeuAcα2-8NeuAcα2-3Galß1-4Glcß1-Cer	nd	0.0	nd	0.0	nd	0.0	15.9	1.8	nd	0.0
619.8/929.6	d18:1	h24:1	NeuAcα2-8NeuAcα2-8NeuAcα2-3Galß1-4Glcß1-Cer	nd	0.0	nd	0.0	nd	0.0	16.3	18.0	nd	0.0
625.6/938.6	t18:0	h24:1	NeuAcα2-8NeuAcα2-8NeuAcα2-3Galß1-4Glcß1-Cer	nd	0.0	nd	0.0	nd	0.0	16.5	10.6	nd	0.0
716.6/1075.1	d18:1	h24:1	NeuAcα2-8NeuAcα2-8NeuAcα2-8NeuAcα2-3Galß1-4Glcß1-Cer	nd	0.0	nd	0.0	nd	0.0	17.4	1.5	nd	0.0
995.6	d18:1	h24:1	Fuc-HexNAc-Galß1-3GalNAcß1-4(NeuAcα2-3)Galß1-4Glcß1-Cer	nd	0.0	nd	0.0	nd	0.0	nd	0.0	15.7	15.1
1003.7	d18:1	h25:1	Fuc-HexNAc-Galß1-3GalNAcß1-4(NeuAcα2-3)Galß1-4Glcß1-Cer	nd	0.0	nd	0.0	nd	0.0	nd	0.0	15.9	11.0
1004.7	t18:0	h24:1	Fuc-HexNAc-Galß1-3GalNAcß1-4(NeuAcα2-3)Galß1-4Glcß1-Cer	nd	0.0	nd	0.0	nd	0.0	nd	0.0	15.9	12.6
1098.2	d18:1	h24:0	HexNAc-(Fuc-)HexNAc-Galß1-3GalNAcß1-4(NeuAcα2-3)Galß1-4Glcß1-Cer	20.1	0.3	nd	0.0	nd	0.0	nd	0.0	nd	0.0
958.7	d18:1	24:1	NeuAcα2-3Galß1-3GalNAcß1-4(NeuAcα2-3)Galß1-4Glcß1-Cer	23.2	2.7	nd	0.0	nd	0.0	15.3	0.7	nd	0.0
966.7	d18:1	h24:1	NeuAcα2-3Galß1-3GalNAcß1-4(NeuAcα2-3)Galß1-4Glcß1-Cer	23.2	1.3	nd	0.0	nd	0.0	nd	0.0	nd	0.0
967.7	d18:1	h24:0	NeuAcα2-3Galß1-3GalNAcß1-4(NeuAcα2-3)Galß1-4Glcß1-Cer	23.0	1.0	nd	0.0	nd	0.0	nd	0.0	nd	0.0
958.7	d18:1	24:1	Galß1-3GalNAcß1-4(NeuAcα2-8NeuAcα2-3)Galß1-4Glcß1-Cer	25.0	3.0	nd	0.0	nd	0.0	nd	0.0	nd	0.0
966.7	d18:1	h24:1	Galß1-3GalNAcß1-4(NeuAcα2-8NeuAcα2-3)Galß1-4Glcß1-Cer	25.6	1.0	nd	0.0	nd	0.0	nd	0.0	nd	0.0
967.7	d18:1	h24:0	Galß1-3GalNAcß1-4(NeuAcα2-8NeuAcα2-3)Galß1-4Glcß1-Cer	25.5	0.7	nd	0.0	nd	0.0	nd	0.0	nd	0.0
1068.1	d18:1	h24:1	HexNAc-Galß1-3GalNAcß1-4(NeuAcα2-8NeuAcα2-3)Galß1-4Glcß1-Cer	nd	0.0	nd	0.0	nd	0.0	17.1	1.5	nd	0.0
1141.2	d18:1	h24:1	Fuc-HexNAc-Galß1-3GalNAcß1-4(NeuAcα2-8NeuAcα2-3)Galß1-4Glcß1-Cer	nd	0.0	nd	0.0	nd	0.0	17.5	0.3	16.4	8.4
1150.2	t18:0	h24:1	Fuc-HexNAc-Galß1-3GalNAcß1-4(NeuAcα2-8NeuAcα2-3)Galß1-4Glcß1-Cer	nd	0.0	nd	0.0	nd	0.0	17.5	0.1	16.3	2.2
1149.2	d18:1	h24:1	Fuc-HexNAc-Galß1-3GalNAcß1-4(NeuGcα2-8NeuAcα2-3)Galß1-4Glcß1-Cer	nd	0.0	nd	0.0	nd	0.0	nd	0.0	16.4	1.0
736.3/1104.6	d18:1	24:1	NeuAcα2-3Galß1-3GalNAcß1-4(NeuAcα2-8NeuAcα2-3)Galß1-4Glcß1-Cer	25.6	1.9	nd	0.0	nd	0.0	nd	0.0	nd	0.0
809.3/1214.1	d18:1	h24:1	NeuAcα2-3HexNAc-Galß1-3GalNAcß1-4(NeuAcα2-8NeuAcα2-3)Galß1-4Glcß1-Cer	nd	0.0	nd	0.0	nd	0.0	18.1	1.6	nd	0.0
926.7	d18:1	14:0	NeuAcα2-3Galß1-3GalNAcß1-4Galß1-3GalNAcß1-4Galß1-4Glcß1-Cer	nd	0.0	nd	0.0	nd	0.0	18.4	0.6	17.3	7.1
1141.2	d18:1	24:1	Galß1-3GalNAcß1-4Galß1-3GalNAcß1-4(NeuAcα2-8NeuAcα2-3)Galß1-4Glcß1-Cer	28.3	0.4	nd	0.0	nd	0.0	nd	0.0	nd	0.0
